# Two Different Clinical Approaches with Mortality Assessment of Four Cases: Complete and Incomplete Type of Abdominal Cocoon Syndrome

**DOI:** 10.1155/2020/4631710

**Published:** 2020-01-29

**Authors:** Ahmet Akbas, Nadir Adnan Hacım, Hasan Dagmura, Serhat Meric, Yüksel Altınel, Ali Solmaz

**Affiliations:** ^1^Department of General Surgery, Bagcılar Training and Research Hospital, Istanbul, Turkey; ^2^Department of General Surgery, Gaziosmanpaşa University, Tokat, Turkey

## Abstract

Abdominal cocoon syndrome (ACS), also called sclerosing encapsulated peritonitis, is a condition characterized by encapsulation of all or some of small bowel loops by a thick fibrous membrane. Etiologic cause is not fully known. It is among the rare causes of intestinal obstruction in adults. Preoperative diagnosis is difficult, and high suspicion is required. Diagnosis is generally made during laparotomy performed due to mechanical obstruction. In treatment of the condition, large scale surgical resections should be avoided. In the present study, we aimed to evaluate all clinical and radiological characteristics and surgical treatment of ACS in light of the literature through four patients operated in our clinic.

## 1. Introduction

Abdominal cocoon syndrome (ACS) is a rare condition causing acute and subacute intestinal obstruction (IO) symptoms. It is caused by a complete or incomplete encapsulation of small bowel loops by a thick fibrous membrane. Manifestation of clinical symptoms is dependent upon the extent of fibrous capsule [1, 2]. It is commonly observed in young women without an operation history. It could develop as idiopathic but could also be caused by secondary reasons which trigger fibrous capsule formation such as past abdominal surgery, tuberculosis, and peritoneal dialysis [3]. Because of its rarity and lack of specific symptoms, diagnosis is usually made during laparotomy. The aim of the treatment is excision of fibrous capsule which leads to fibrosis and relieving of IO [1, 3].

The aim of the present study was to evaluate the clinical and radiological findings of four patients diagnosed with ACS who were operated with mechanic IO prediagnosis in light of the literature.

## 2. Case Reports

### 2.1. Case 1

**Figure 1:**
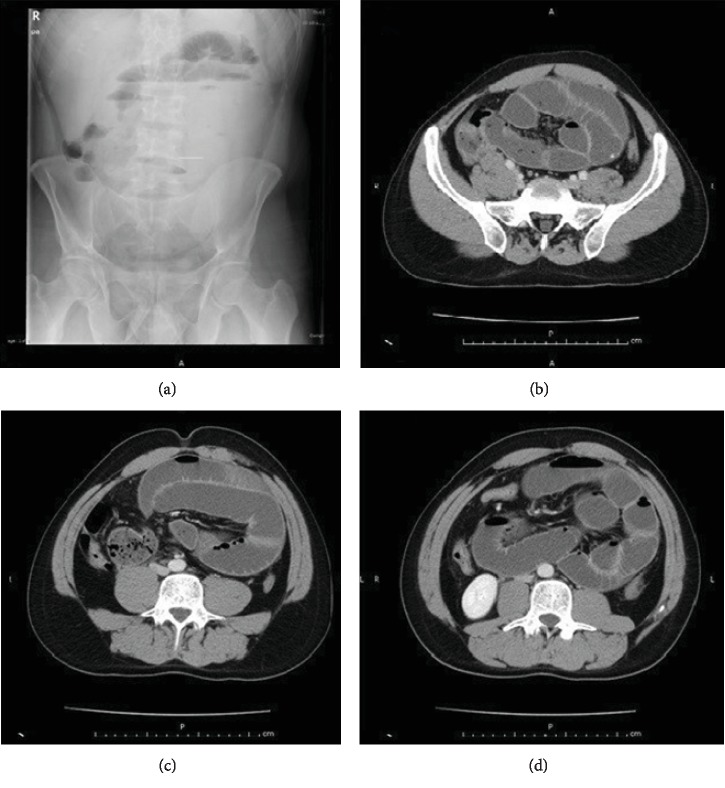
Case 1: air-fluid levels on X-ray showed an obstruction in the small intestine (a). Coronal sections of abdominal CT images indicated dilatation and thickening of the small intestine wall (b–d).

A 30-year-old male patient with nausea, vomiting, and abdominal pain complaints lasting for a few days presented to our clinic. In his anamnesis, he had several previous abdominal pain attacks which healed themselves. He had not had any abdominal surgery or medicine use before. Clinical examination revealed a tender and distended abdomen. Plain X-ray of the abdomen showed air-fluid levels of small bowel type. On the other hand, abdominal CT revealed dilatation and wall thickness in the terminal ileum (Figure 1). No abnormality was observed in laboratory parameters. An exploratory surgery with a laparotomy method was decided with “acute mechanical intestinal obstruction” prediagnosis. During the laparotomy, a cocoon-like fibrous tissue of about 20 cm diameter was observed around the ileum. Small bowel was relieved through cutting the fibrous membrane. Since the circulation was normal in bowel segment, no resection was made. The patient was discharged on the fourth day after the operation.

### 2.2. Case 2

**Figure 2:**
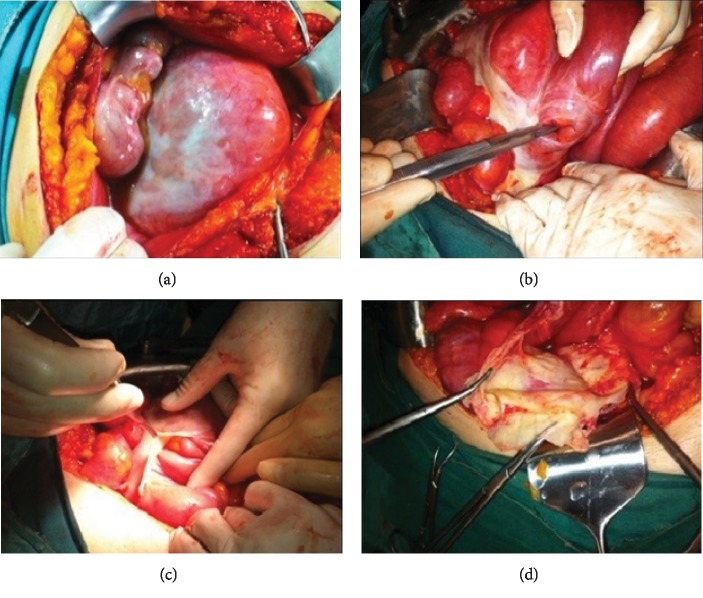
Case 2: encapsulated part of the small intestine (a). Loosening of encapsulated segments (b, c). Appearance of fibrous capsule after decapsulation (d).

A 47-year-old male patient presented to our hospital with the complaints of constipation, nausea, and vomiting for three days. No significant medical or clinical record was apparent in his medical past, including any previous abdominal operations or medication use. Clinical examination revealed signs of only abdominal distension. Plain abdominal X-ray indicated air-fluid levels while abdominal CT showed proximally dilated clustered terminal ileum and cecum. Laboratory parameters were normal, and exploratory laparotomy was carried out. During laparotomy, a cocoon-like fibrotic tissue with a diameter of about 10 cm was found to surround internally herniated ileum and cecum (Figure 2). Bowel loops were relaxed after cutting the fibrous structure. Since the circulation was normal in the affected bowel segment, resection was not made during the laparotomy. The patient was discharged five days after the operation with no complications.

### 2.3. Case 3

**Figure 3:**
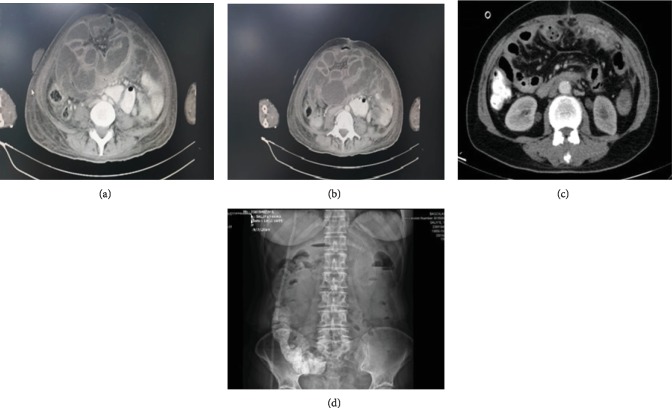
Computed tomography and plain abdominal X-ray of the patients with complete type 3 ACS. Case 3: (a) and (b). Case 4: (c) and (d).

**Figure 4:**
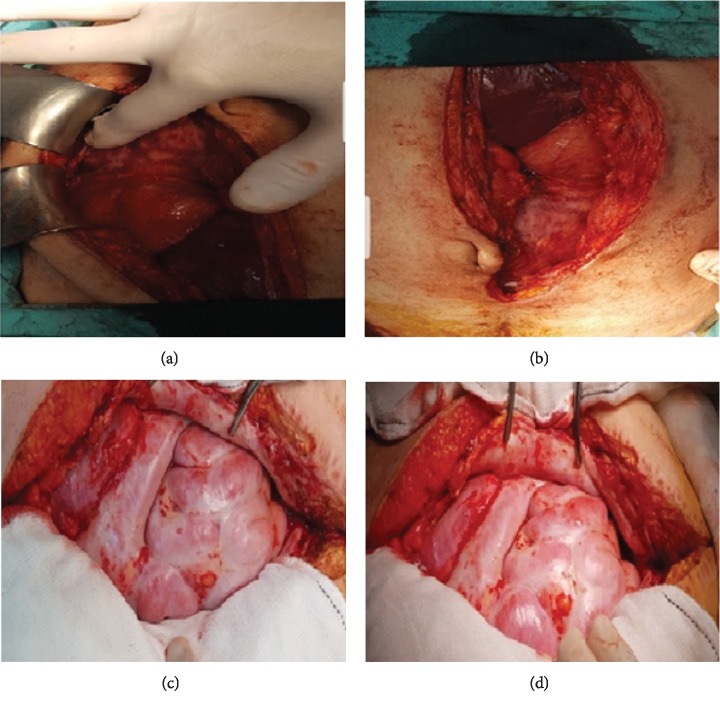
Laparotomy appearance of the patients with complete type 3 ACS. Case 3: (a) and (b). Case 4: (c) and (d).

A thirty-eight-year-old female patient applied to our hospital with the complaints of swelling, vomiting, and constipation for a week. She had sigmoid colon tumor diagnosis two months ago and underwent left hemicolectomy. Clinical examination revealed only distended abdomen. Plain X-ray of the abdomen indicated air-fluid levels while abdominal CT showed clustering of proximally dilated ileum and cecum. Laboratory findings were normal (Figures 3(a) and 3(b)). Exploratory laparotomy showed pronounced adhesion in the abdomen. It was observed that small bowels, colon, stomach, and liver were adhered as surrounded en bloc by a fibrous capsule in a way that anatomic boundaries could not be distinguished (Figures 4(a) and 4(b)). No area was observed for adhesiolysis or resection in the patient diagnosed to have ACS. Due to continuous vomiting and lack of oral intake, percutaneous gastrostomy (PG) was applied for palliative purposes. Abdominal fascia was extremely rigid and not suitable for closing. The skin was closed with primary suture, and the patient was monitored in surgical oncology department in postoperative period. The patient became ex on the 37^th^ postoperative day.

### 2.4. Case 4

A sixty-four-year-old female patient applied to our hospital with the complaints of abdominal swelling, vomiting, and constipation which had been continuing for three days. Her history revealed TAH+BSO+pelvic and para-aortic lymph node dissection due to endometrium cancer a month ago. Clinical examination showed only abdominal distension. No feature was observed in plain abdominal X-ray and abdominal CT examinations. Laboratory parameters were normal (Figures 3(c) and 3(d)). Exploratory laparotomy was decided for the patient. Advanced level adhesion was observed in the abdomen during laparotomy. The small bowels, colon, stomach, and liver were adhered and surrounded en bloc by a fibrous capsule in a way that anatomic boundaries could not be distinguished (Figures 4(c) and 4(d)). No area was found appropriate for adhesiolysis or resection in this ACS patient. Percutaneous gastrostomy (PG) was applied to the patient with palliative purposes because of her continuous vomiting and lack of oral food intake. Abdominal fascia was extremely rigid and was not suitable for closing. The skin was closed with primary suture. The patient became ex on the 25^th^ postoperative day.

## 3. Discussion

ACS, also known as abdominal cocoon, sclerosing encapsulated peritonitis, primer sclerosing peritonitis, or idiopathic sclerosing peritonitis, was first described by Hu et al. and Kanat et al. [2, 4]. It is a condition in which a thick fibrous tissue partly or completely encapsulates small bowels and leads to decreased motility of small bowel. It is characterized by peritoneal inflammation. Fibrous capsule could sometime form an adhesion through encapsulation all abdominal organs in addition to small bowels [5]. It is one of the rare causes of abdominal obstruction, and its etiology is unknown. Peritoneal irritation and/or inflammation due to any etiological agent is considered to induce peritoneal fibrogenesis [6]. ACS has two forms: idiopathic and secondary. Idiopathic ACS is due to congenital reasons, and no acquired cause is involved in cocoon formation. Idiopathic form is usually observed as accompanied to embryological anomalies such as greater omentum hypoplasia and mesenteric vessel malformation. Secondary form, on the other hand, could arise as a result of immunological diseases (SLE and sarcoidosis), infections (tuberculosis), cirrhosis, liver plantation, ventriculoperitoneal shunts, chronic ambulatory peritoneal dialysis, intravenous medicine administration, gastrointestinal malignancies, intra-abdominal surgery (abdominal washing using povidone), beta-adrenergic blocker use (proctolol), familial Mediterranean fever, protein S failure, and gynecological diseases (uterus leiomyoma, endometriotic cysts, luteinized ovarian thecoma, and ruptured dermoid cyst) [4, 7–9]. Various acquired factors play a role in its etiology, but the common feature is the development of inflammatory reaction inside the abdomen [1].

Some publications categorize ACS in three classes based on the affected organ. In type 1 ACS, a small section of small bowel is surrounded by fibrous capsule. In type 2, all of small bowels are encapsulated by fibrous capsule, and in type 3, all small bowels and other organs are encapsulated by fibrous capsule [10, 11]. ACS was due to idiopathic causes in two of our cases, while in two patients, it occurred due to secondary causes (recent surgical treatment). In two ACS with idiopathic origin, small bowels were segmentally encapsulated by fibrous capsule (type 1). In two ACS which developed due to secondary causes, small bowels, colon, and stomach were encapsulated en bloc in the form of a cocoon within the abdomen. Occurrence of clinical findings in ACS could be acute, subacute, or chronic. Acute indications are colic pain, vomiting, constipation, and distension in the abdomen, while chronic symptoms are abdominal tension, changes in bowel habits, sustained mild abdominal pain, and less frequently anorexia and weight loss [2, 6, 12]. Yip and Lee [13] described four diagnostic features that could help in ACS diagnosis: (1) nausea, vomiting, weight loss, abdominal distension, and current pain attacks due to acute and subacute small bowel obstruction; (2) palpation of nontender mass in abdominal palpation; (3) typical radiological appearance; and (4) lack of a previous abdominal operation history [6, 12]. In physical examination of ACS patient, an asymmetric distension which does not move with peristalsis due to fibrous capsule surrounding small bowels is observed in the inspection of abdominal area. Besides in palpation of abdominal wall, enlarged bowel in the proximal of the adhered section is palpated soft while the obstructed zone is palpated as a rigid, flat, and painless area due to fibrous tissue [14, 15]. None of our patients had clinical or radiological findings indicative of ACS in preoperative period. In physical examination of two patients with type 3 ACS, an 8 × 10 cm intra-abdominal rigid mass could be palpated around the belly. In all four patients, ACS diagnosis was made during laparotomy. Correct preoperative diagnosis is difficult in ACS due to lack of specific clinical, laboratory, and radiological methods. Air-fluid levels could be observed in plain abdominal X-ray due to intestinal obstruction, but they are not specific to ACS. Seick defined classical barium X-ray findings as delaying in contrast matter passage, U-shaped clustering of small bowel loops which have an appearance of accordion- or a cauliflower-shaped appearance. Nonetheless, these findings are not always observed, and they are nonspecific [6, 12]. USG findings of ACS are variable and include hanging of bowel on posterior abdominal wall, dilatation and fixation of small bowel loops, a thick-walled mass containing intestinal loops, and loculated ascites and fibrous adhesions. Since the small bowel loops are enlarged in ACS, USG has only a limited significance in ACS diagnosis. Compared to other imaging methods, CT provides more information and helps in ruling out the other intestinal obstruction causes. Observation of peritoneal calcification, thickening in peritonea, loculated liquid collections, small bowel loops clustered in a single area along with sac-like clustered small bowel loops within a thin membrane, thickened intestinal wall, and presence of a thick fibrous capsule surrounding spleen and liver are diagnostic features of ACS. However, these features may not be observed in CT of all ACS patients [3, 12, 16] (Table 1). CT revealed no finding pointing to ACS in any of our cases. Ileus findings were observed in three patients, while in the other patient (Case 4), no feature was observed in plain abdominal X-ray and CT (Figures 4(a) and 4(c)). ACS is treated by removing the fibrous capsule. When adhesiolysis could not be performed, resection anastomosis could be tried as an alternative treatment modality (Table 1). It is important to keep the resected segment at minimum. The aim of the surgery is to achieve the passage through removal of the section causing intestinal obstruction [17]. In patients of the present study, treatment was performed through removing the fibrous capsule in two patients while the other two patients had complete type 3 ACS, and thus, no section was found suitable for adhesiolysis or resection in these patients. Due to their persistent vomiting which already existed in preoperative period, PG was applied to them for nutritional or drainage purposes when the vomiting was excessive.

## 4. Conclusion

ACS is a condition in which small bowel is partly or completely enclosed by a fibrous capsule in adults. It is among the rare causes of mechanical intestinal obstruction. The disease could develop with acute or subacute manifestations. Preoperative diagnosis is difficult, and diagnosis is generally made during laparotomy. In patients with incomplete type of ACS, it can be possible to achieve complete healing. On the other hand, short-term mortality is common in the cases with complete type of the disease. ACS should be considered in the differential diagnosis of patients who presented with acute or subacute ileus attacks.

## Figures and Tables

**Table 1:** Some case reports of ACS in the literature.

Number	Authors and year	Age	Gender	Preoperative diagnosis	Diagnostic tool	Intraoperative findings
1	Akbulut, 2015 [1]	87	M	ACS	CT and surgery	Encapsulation of part of small bowel
2	Hu et al., 2013 [2]	29	F	Int. Obst.	Surgery	Encapsulation of all small bowel
3	Yip and Lee, 1992 [13]	30	M	ACS	CT and surgery	Encapsulation of part of small bowel
4	Yeniay et al., 2011 [14] (two cases)	71	M	Int. Obst.	Surgery	Encapsulation of part of small bowel
		26	F	Int. Obst.	Surgery	Encapsulation of part of small bowel
5	Oymacı et al., 2016 [15]	32	M	Int. Obst.	Surgery	Encapsulation of part of small bowel
6	Sharma et al., 2013 [16]	42	M	ACS	CT and surgery	Encapsulation of all small bowel
7	Çağlar et al., 2015 [17]	36	F	Int. Obst.	Surgery	Encapsulation of all small bowel

CT: computed tomography; Int. Obst.: intestinal obstruction; ACS: abdominal cocoon syndrome.
